# Supplementary material for: Copy Number Variations and Expression Levels of Guanylate-Binding Protein 6 Gene Associated with Growth Traits of Chinese Cattle
Source: Animals (Basel). 2020 Mar 27;10(4):566. doi: 10.3390/ani10040566 (PMC7222342; doi:10.3390/ani10040566)
Supplement: Supplementary file 1 [file animals-10-00566-s001.pdf]

Supplementary materials

Supplementary table 1. Relative expression values of *GBP6* in seven tissues of QC and XN cattle in fetal, calf and adult stages with three replicates.

Breed	Stage	Tissue	Relative expression value		
			Replicate 1	Replicate 2	Replicate 3
QC	Fetal	Heart	2.94	3.01	3.16
QC	Fetal	Liver	9.75	4.33	16.05
QC	Fetal	Spleen	13.17	10.57	13.60
QC	Fetal	Lung	4.36	3.08	8.42
QC	Fetal	Kidney	5.72	7.06	13.07
QC	Fetal	Muscle	9.21	12.99	18.56
QC	Fetal	Fat	0.00	3.21	5.25
QC	Calf	Heart	7.98	8.02	7.82
QC	Calf	Liver	3.64	3.72	3.95
QC	Calf	Spleen	7.63	9.71	14.54
QC	Calf	Lung	6.87	9.88	20.86
QC	Calf	Kidney	13.94	4.35	16.40
QC	Calf	Muscle	2.02	0.13	10.67
QC	Calf	Fat	12.29	4.61	9.79
QC	Adult	Heart	6.37	6.43	6.62
QC	Adult	Liver	3.62	1.97	0.85
QC	Adult	Spleen	33.99	35.17	54.75
QC	Adult	Lung	16.14	9.83	7.32

QC	Adult	Kidney	4.12	2.51	3.77
QC	Adult	Muscle	1.68	1.00	0.68
QC	Adult	Fat	11.65	9.53	9.05
XN	Adult	Heart	2.77	2.86	2.86
XN	Adult	Liver	2.04	1.60	0.92
XN	Adult	Spleen	43.44	34.72	40.80
XN	Adult	Lung	23.99	29.35	26.41
XN	Adult	Kidney	11.54	6.59	13.60
XN	Adult	Muscle	0.55	7.95	13.98
XN	Adult	Fat	4.67	2.29	5.23

Note: NY: Nanyang breed; QC: Qinchuan breed; XN: Xianan breed.
